# CD200 as a Potential New Player in Inflammation during Rotator Cuff Tendon Injury/Repair: An In Vitro Model

**DOI:** 10.3390/ijms232315165

**Published:** 2022-12-02

**Authors:** Raffaella Giancola, Francesco Oliva, Marialucia Gallorini, Noemi Michetti, Clarissa Gissi, Fadl Moussa, Cristina Antonetti Lamorgese Passeri, Alessia Colosimo, Anna Concetta Berardi

**Affiliations:** 1 Department of Haematology, Transfusion Medicine and Biotechnologies, Cytofluorimetry and Cell Sorting Service, Ospedale Spirito Santo, 65122 Pescara, Italy; 2 Department of Musculoskeletal Disorders, Faculty of Medicine and Surgery, University of Salerno, 84084 Baronissi, Italy; 3 Clinica Ortopedica, Ospedale San Giovanni di Dio e Ruggi D’Aragona, 84131 Salerno, Italy; 4 Department of Pharmacy, University G. d’Annunzio, 66100 Chieti, Italy; 5 Department of Medicine, University of Udine, 33100 Udine, Italy; 6 Faculty of Bioscience and Technology for Food, Agriculture and Environment, University of Teramo, 64100 Teramo, Italy; 7 Doctoral School of Science and Technology, Lebanese University, Beirut 1107, Lebanon; 8 Department of Haematology, Transfusion Medicine and Biotechnologies, Laboratory of Stem Cells, Ospedale Spirito Santo, 65122 Pescara, Italy

**Keywords:** tendons, rotator cuff disease, inflammation, CD200, TNFα, IFNγ, tendon stem cells, CD146, *IRF1*, *C* */EBPbeta*, DOK2

## Abstract

Rotator cuff tendon (RCT) disease results from multifactorial mechanisms, in which inflammation plays a key role. Pro-inflammatory cytokines and tendon stem cell/progenitor cells (TSPCs) have been shown to participate in the inflammatory response. However, the underlying molecular mechanism is still not clear. In this study, flow cytometry analyses of different subpopulations of RCT-derived TSPCs demonstrate that after three days of administration, TNFα alone or in combination with IFNγ significantly decreases the percentage of CD146+CD49d+ and CD146+CD49f+ but not CD146+CD109+ TSPCs populations. In parallel, the same pro-inflammatory cytokines upregulate the expression of CD200 in the CD146+ TSPCs population. Additionally, the TNFα/IFNγ combination modulates the protein expression of STAT1, STAT3, and MMP9, but not fibromodulin. At the gene level, *IRF1*, *CAAT (CAAT/EBPbeta)*, and *DOK2* but not *NF-κb*, *TGRF2 (TGFBR2)*, and *RAS-GAP* are modulated. In conclusion, although our study has several important limitations, the results highlight a new potential role of CD200 in regulating inflammation during tendon injuries. In addition, the genes analyzed here might be new potential players in the inflammatory response of TSPCs.

## 1. Introduction

Tendon healing after an acute injury is an ineffective process, rarely restoring complete mechanical functionality of the damaged tissue. Studies suggest that the early inflammatory response during the first stage of tendon healing plays a crucial role in the onset and progression of tendinopathy [1,2,3]. Indeed, the enhanced expression of pro-inflammatory cytokines and the consequent persistent inflammatory response has been linked to tendinopathy [2]. However, the role of sustained cytokine signaling under inflammatory conditions in the development, progression, and resolution of tendon injuries remains controversial [4,5].

Resident tendon stem/progenitor-cells (TSPCs) represent 1% to 4% of the total tendon cell population and express a cluster of differentiation (CD)146, CD90, and CD44 [6,7], as well as tenocyte-specific markers, such as scleraxis (Scx) [8].

Although it has been shown that cytokines secreted at the injured site during inflammation affect resident TSPCs, which regulate tendon repair through the c-Jun N-terminal kinase (JNK)/signal transducer and activator of transcription 3 (STAT3) signaling pathways, most of the mechanisms are still unclear [9,10]. The glycoprotein CD200 type-1, belonging to the immunoglobulin supergene family, is one of several cell transmembrane proteins playing an active role during inflammation. Recently, CD200 was found to inhibit immune responses by engaging the CD200 inhibitory receptor (CD200R1), whose expression is restricted to myeloid-derived antigen-presenting cells (APCs) and some T-cell populations [11,12,13,14]. CD200 expression can be induced by pro-inflammatory cytokines, such as TNF-α and IFN-γ, in an NF-kB-, STAT1-, and IRF-1-dependent manner [14,15]. Interestingly, CD200 is expressed in several tissues/cells, including CD146+ stem cells originating from the Achilles and patellar tendons [16]. However, there are no data for CD200 expression and cytokine response in rotator cuff-derived tendon cells (RCTCs). 

Moreover, given the ability of stem/progenitor cells from other sources to modulate inflammation [7,17], the role of TSPCs in the process of tendon inflammation deserves further investigation.

The aim of the present study was to analyze in vitro response of RCTCs to TNFα and IFNγ pro-inflammatory cytokines. We herein report the effect of pro-inflammatory cytokines on TSPCs surface-markers expression, as well as on the mRNA and protein levels of selected target genes that are involved in inflammation. 

## 2. Results

### 2.1. Identification of Cell Surface Markers Characterizing the TSPC Population

**Figure 1:**
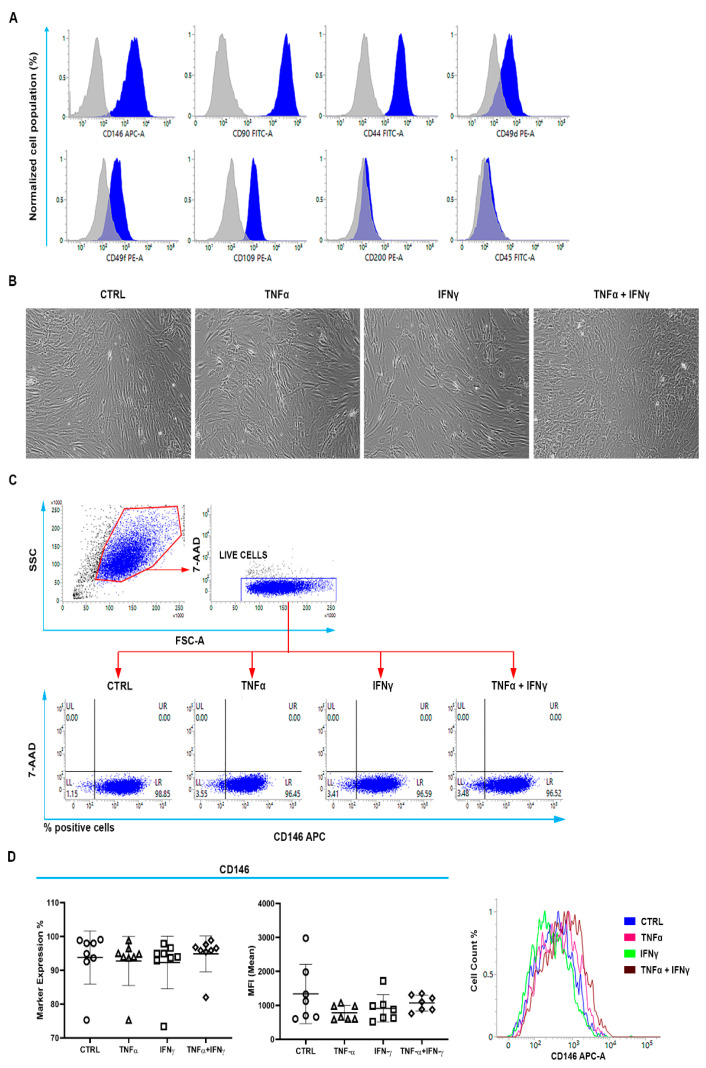
Immunophenotypic profile of human rotator-cuff-tendon-derived cells (RCTCs) by flow cytometry. (**A**) Cells were stained for a panel of the cluster of designation (CD): CD146, CD90, CD44, CD49d, CD49f, CD109, CD200, and CD45. Peaks of fluorescence emission were obtained by flow cytometry, and their right-shifted peak (blue) represents the positivity for the marker analyzed with respect to the isotype negative control (grey peak). CD146 (74% ± 16.1), CD90 (99% ± 0.3), CD44 (100% ± 0.4), CD49d (58% ± 26.6), CD49f (74% ± 8.8), and CD109 (73% ± 19.8). (**B**) Microscopic analysis of cell morphology after 3 days of in vitro stimulation of proinflammatory cytokines. Representative images of cells exposed to treatments were acquired by phase-contrast microscopy. 100× magnification. (**C**) Gating strategy. The SSC (side scatter)/FSC (forward scatter) dot plot allows the gating of the cell population by means of its morphological parameters. Cells were afterward stained with the 7-AAD (7-aminoactinomycin) to exclude dead cells from further analyses (CD146). No altered expression of CD146 was promoted after stimulation with single or combined pro-inflammatory cytokines. (**D**) Graphs represent the percentage of CD146 expression and the MFI (mean fluorescence intensity) of cells exposed to treatments. Fluorescence emission peaks related to CD146 were obtained by flow cytometry. CTRL = untreated cells.

Since we found that RCTCs modify their antigen expression during serial passaging (P), especially after P5, we only used cells at P2 for reproducible outcomes. To identify a TSPC subpopulation in isolated RCTCs, we found that most cells expressed high levels of CD146, CD90, CD44, and known TSPC markers [7,8]. Moreover, we found low expression of α4 and α6 integrins (CD49d, CD49f) and a medium expression of glycophosphatidylinositol-anchored protein CD109, which is known to bind and regulate transforming growth factor-beta (TGF-beta) signaling. Interestingly, we found low expression of CD200 and no expression of CD45, which was consistent with the connective tissue origin of tendon-derived cells (Figure 1A). Our data confirm the presence of a subpopulation of TSPCs (named CD146+TSPCs) [6,7] in isolated RCTCs expressing low basal surface CD200.

### 2.2. CD146+TSPCs Response to TNFα and IFNγ

To analyze the effects of TNFα and IFNγ stimulation on CD146+TSPCs in the RCTCs, we treated RCTCs with TNFα or IFNγ or both for 3 days. We observed RCTCs morphology changing from a spindle shape to a rather roundish one, only with the combination of TNFα and IFNγ, and not with either cytokine alone (Figure 1B). Neither single nor combination cytokine treatment altered CD146 marker expression in TSPCs (Figure 1C,D).

### 2.3. TNFα and IFNγ Increased the Expression of CD146+CD200+ TSPCs

**Figure 2:**
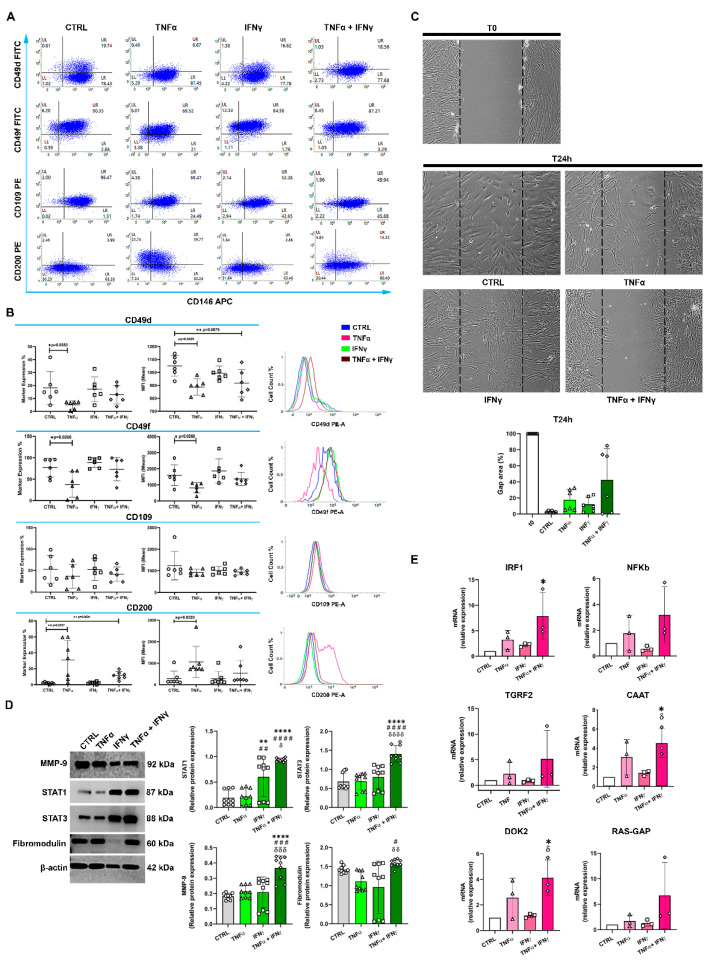
Analysis of inflammation markers in the CD146+TSPCs population. (**A**) RCTCs, stained positive for CD146, were afterward co-stained for the cluster of designation (CD)49d, CD49f, CD109, and CD200. Representative dot plots show the distribution of the cell population in response to treatments. (**B**) Graphs represent the percentage of marker expression and the MFI (mean fluorescence intensity) related to CD49d, CD49f, CD109, and CD200. Relative emission peaks were obtained by flow cytometry. The right shift of peaks represents a higher positivity for markers. (**C**) Migration of RCT-derived cells in response to the various treatments immediately after the stimulus (T0) and after 24 h. Representative images obtained by phase-contrast microscopy. The bar graph represents the percentage of cells covering the gap (empty) area. 100× magnification. (**D**) Protein expression of MMP (metalloproteinase)-9, STAT (Signal transducer and activator of transcription)1, STAT3, and fibromodulin detected by Western blotting after 24 h. β-actin is used as a loading control. Bar graphs display densitometric values normalized on the ones of the loading control (relative expression). (**E**) Graphs represent relative gene expressions of *IRF* (Interferon Regulatory Factor)-1, *NFkb* (Nuclear factor kappa subunit b), *TGRF2* (*TGFBR2*-Transforming growth receptor factor 2), *CAAT* (CAAT/Enhancer-binding protein beta), *DOK2* (docking protein 2), and *RAS-GAP* (Ras GTPase activating protein). * = *p* < 0.05; ** = *p* < 0.01; and **** = *p* < 0.0001 between CTRL and treated cells. # = *p* < 0.05; ## = *p* < 0.01; ### = *p* < 0.001 and #### = *p* < 0.0001 between cells treated with TNFα and cells in the presence of other treatments. δ = *p* < 0.05; δδ = *p* < 0.01; δδδ = *p* < 0.001 and δδδδ = *p* < 0.0001 between cells treated with IFNγ and cells in the presence of other treatments.

The percentage of CD146+CD49d+ cells decreased significantly after TNFα stimulation (*p* ≤ 0.04), but did not change after IFNγ administration (Figure 2B), and decreased only slightly with combination treatment. Analogously, the MFI values for CD146+CD49d+ cells decreased significantly with TNFα alone (*p* ≤ 0.05) or TNFα in combination with IFNγ (*p* ≤ 0.01), but not with IFNγ alone (Figure 2B). The percentage of CD146+CD49f+ cells decreased significantly with TNFα (*p* ≤ 0.03), increased moderately with IFNγ alone, and remained largely unchanged with the TNFα/IFNγ combination. A similar trend was observed in the corresponding MFI values. TNFα or TNFα+IFNγ did not significantly affect the proportion of CD146+ CD109+ TSPCs (Figure 2A,B). Finally, a significant increase in the percentage of the CD146+CD200+ cell population was observed upon TNFα stimulation (*p* ≤ 0.0017) and combination TNFα/IFNγ treatment (*p* ≤ 0.01) but not with IFNγ alone (Figure 2A). MFI values for this cell subpopulation showed analogous results, with a significant increase only after TNFα stimulation (*p* ≤ 0.032) (Figure 2B).

### 2.4. The In Vitro Gap Repair Assay

To measure the repair capacity of RCTCs, we used an in vitro gap repair assay. As shown in Figure 2C, 24 h treatment of RCTCs with cytokine alone or in combination showed a non-significant decrease in cell migration compared to the unstimulated cells. Notably, RCTCs treated with the TNFα/IFNγ combination showed slower gap closure.

### 2.5. IFNγ+TNFα Increased the Expression of STAT1, STAT3, and MMP9 Proteins

We then measured downstream signaling in response to cytokine stimulation. While TNFα did not significantly increase STAT1 expression in RCTCs, IFNγ significantly increased it (*p* ≤ 0.001), while the TNFα/IFNγ combination increased STAT1 levels even more (*p* ≤ 0.0001). Both STAT3 and MMP9 protein levels were significantly increased only with the TNFα/IFNγ combination (*p* ≤ 0.0001) (Figure 2D), while neither cytokine nor the combination of them significantly affected fibromodulin levels (Figure 2D).

### 2.6. IFNγ+TNFα Increased the Expression of IRF1, CAAT and DOK2 mRNA

*IRF1* mRNA expression was not significantly modulated in RCTCs with TNFα and IFNγ alone, but it increased significantly with combination treatment (*p* ≤ 0.05). On the other hand, *NF-κB* and *TGFR2* mRNA levels were not significantly modulated by IFNγ+TNFα. The levels of *CAAT* and *DOK2* mRNA increased slightly with TNFα, but not with IFNγ, but increased significantly with combination treatment (*p* ≤ 0.05) (Figure 2E). *RAS-GAP* mRNA expression was not significantly modulated by cytokines in RCTCs.

## 3. Discussion

The role of inflammation in tendon injury/repair remains poorly understood. This study shows that RCTCs contain a population expressing CD146, CD90, and CD44 TSPC surface-markers. Additionally, they co-express CD49d (integrinα-4); CD49f (integrinα-6), a known and specific stem-cell population marker [18,19]; CD109; and, interestingly, the CD200 ligand. In vitro stimulation on RCTCs using pro-inflammatory cytokines TNFα and IFNγ only revealed significant modulation of CD146+CD49d+ and CD146+CD49f+ expression using TNFα alone. This result likely indicates CD146+TSPC activation, which may influence recruitment and survival and, for CD49f, self-renewal regulation in TSPCs, as previously reported [19,20]. However, migration results showed no significant difference for 24 h pro-inflammatory cytokine stimulation in in vitro culture, compared to the control. Noticeably, migration capacity for the whole RCTC population decreased using TNFα and IFNγ in combination. These findings, together with morphological observations, suggest that the above cytokines may induce biochemical and molecular cellular changes, thus requiring further studies. In previous research, CD109 inhibition suppressed inflammation, by reducing pro-inflammatory factor production, cell migration, invasion, chemo-attractive potential, and osteoclast differentiation [21]. Here, CD109 expression was not modified by pro-inflammatory cytokines. A novel finding was that TNFα alone or TNFα+IFNγ significantly increased CD200 marker expression in the CD146+TSPCs. Similar results have been found in mesenchymal stem/stromal cells [22]. Previous studies highlighted a fundamental regulatory role in controlling inflammation for the CD200 ligand interacting with CD200R [11,12,13,14,15]. Our results suggest an active role for TSPCs in regulating inflammatory processes during tendon injury/repair, through the interaction of CD200, expressed on CD146+TSPCs, with CD200R, located on immune-competent cells. STAT1 and STAT3, members of the cytoplasmic family of transcription-factor (STAT) signal-transducers and activators, have been associated with inflammatory pathologies, including tendinopathy [23].

Our study clearly demonstrates that co-administration of TNFα and IFNγ induces a significant increase in STAT1 and STAT3 protein levels. These results agree with previous research showing crosstalk between TNFα and IFNγ signaling pathways and suggest the molecular control of STAT1 availability to tumor necrosis factor receptor 1 (TNFR1) [24]. STAT1 and STAT3 are known to play antagonistic roles and disruption of their balanced interaction redirects cells from survival to apoptotic death, or from inflammatory to anti-inflammatory response [25]. Most importantly, STAT3 has been shown to play a key role in healing tendons [9]. TNFα and IFNγ have been reported to affect metalloproteinase (MMP) synthesis, and their ability to upregulate MMP9 expression leads to matrix destruction and remodeling [6,26]. Accordingly, our data show a significant increase in MMP9 protein levels after TNFα and IFNγ co-stimulation. Proteoglycan fibromodulin, a critical component of the ECM involved in collagen assembly and tendon repair [27], was not modulated by TNFα and IFNγ in our study.

TNFα and IFNγ have been shown to induce the expression of *IRF1* (ubiquitously expressed in human cells), associated with STAT pathway activation [28,29,30,31]. Additionally, increased *IRF1* expression is also found in tendinopathy [32]. Accordingly, our results indicate that TNFα and IFNγ together induce a significant increase in *IRF1* mRNA in RCTCs. *NF-κB*, which has already been shown to play a role in inflammation, is activated by pro-inflammatory cytokines, including TNFα and IFNγ. *NF-κB* expression is also dependent on IRF1 activation, and increased *NF*-*κ*B levels are detected in early RC tendinopathy [33,34,35,36]. In our study, *NF*-*κ*B was not significantly modulated by TNFα and IFNγ cytokines. Similarly, no significant modulation of *TGFR2* (*TGFBR2*) was observed in our in vitro model, although knockout of the *TGFBR2* gene in tenocytes has been shown to attenuate development of tendinopathy [37].

The activity and expression levels of CAAT/Enhancer-binding protein beta (*C/EBPbeta*), involved in the maintenance of normal function and response to injury, are regulated by several inflammatory agents, including TNFα and IFNγ [38]. Here, for the first time, we demonstrate that co-administration of TNFα and IFNγ significantly modulates *CAAT* mRNA expression in RCTCs. Furthermore, DOK2, which may have a role in various physiological functions, including both innate and adaptive immunities, could also act as a negative regulator of cell proliferation when stimulated by cytokines [39]. Accordingly, we have shown the significant modulation of *DOK2* mRNA after TNFα and IFNγ stimulation in RCTCs. These results suggest a possible role for *DOK2* in tendinopathy. Finally, we investigated *RAS-GAP* mRNA expression after pro-inflammatory cytokine administration in RCTCs, since it is involved in many aspects of cell biology. In our in vitro study, *RAS-GAP* mRNA was not significantly modulated by TNFα and IFNγ in RCTCs.

Our study has the following limitations: (1) tendon repair, in vitro or in vivo, using KO or over expression approaches, should be analyzed in order to infer any “potential” mechanistic role of one or the other markers (CD200 or others); (2) to determine the potential involvement of CD200 in reduced cell migration in cells treated with two cytokines, it would be better to use lentivirus and see whether this would affect the phenotype; (3) rather than performing qRT-PCR on a few selected markers, it would be potentially more interesting to perform RNAseq analysis, which could lead to the identification, potentially, of previously unknown targets; (4) further research is needed to explain why if both STAT1 and STAT3 are upregulated and how they can have antagonistic activities. Despite these considerations, this study enhances the understanding of RCTC populations in inflammatory conditions, including stem/progenitor subpopulations, and suggests an important role for CD200 among the various markers. Further studies are necessary to evaluate the role of genes whose mRNA expression is increased by TNFα and IFNγ, such as *IRF1*, *C/EBPbeta*, and *DOK2*, and to deeply understand how CD200 activation may regulate inflammation. Identifying underlying molecular mechanisms may provide the basis for the development of innovative therapies for RC tendinopathy.

## 4. Materials and Methods

### 4.1. Rotator Cuff Tendon-Derived Cells Cultures

RCTCs that were isolated from the same 10 patients described in our previous work [40] and cryopreserved in liquid nitrogen were used. The isolation protocol was described previously [40,41,42]. The cell phenotype was confirmed by assessing the expression of a tenocyte-specific gene (scleraxis) and genes for collagens α1(I), α2(I), and α1(III) by real-time PCR, as previously described (not shown) [43].

For the present study, cells at passage 0 were thawed out and sub-cultured in alpha-MEM with 10% heat-inactivated FBS and 1% penicillin/streptomycin (Gibco, MA, USA) at 37 °C and 5% CO_2_. Cells at passage 2 (P2) were used to avoid phenotypic drift [44], were seeded at 1.5 × 10^5^ cells/flask in a 25 cm^2^ culture flask, and were allowed to adhere overnight. Afterward, cells were exposed to complete alpha-MEM (untreated control) or stimulated by cytokines at a final concentration of 10 ng/mL as previously described [45]. In detail, TNFα alone, IFNγ alone, or IFNγ and TNFα in combination (PeproTech, London, UK) were added to the medium.

### 4.2. Flow Cytometry

RCTCs were stained with a panel of fluorochrome-conjugated, monoclonal antibodies: CD45-FITC, CD90-FITC, CD49d-PE, CD49f-PE, CD109-PE, (BD Pharmingen, San Diego, CA, USA) CD44-FITC, CD146-APC, and CD200-PE (Miltenyi Biotech, Bergisch Gladbach, Germany). Cells were acquired with a BD FACSLyric II flow cytometer (BD Biosciences, CA, USA) equipped with a 488 nm, 640 nm, and 405 nm laser. Events were analyzed using Suite1.5 and FlowJo 10.6.2 software (BD Biosciences). Results are shown as cell positivity percentages or as mean fluorescence intensities (MFIs).

### 4.3. In Vitro Gap Repair Assay

Cells were grown to confluence in 24-well plates, and the scratch was made using a sterile P10 pipette tip, creating a cell-free area, as described before [46]. Cultures were treated in reduced FBS conditions (1% FBS) and images were acquired immediately after wounding (T0) and after 24 h through bright field microscopy (NIKON, Melville, NY, USA). Images were analyzed by ImageJ (Version 1.49 v, RRID:SCR_003070; NIH, Bethesda, MD, USA), and cell-free areas were marked; outcomes are represented as a percentage of the initial wound area.

### 4.4. Immunoblotting

At 24 h, cells were harvested and lysed as previously reported [42]. Twenty micrograms of whole-protein fraction were loaded on a 12% sodium dodecyl sulfate-polyacrylamide gel followed by Western blot. Nitrocellulose membranes were blocked and probed overnight at 4 °C with mouse monoclonal anti-STAT1 and anti-STAT3, rabbit polyclonal anti-fibromodulin (1:1000; Abcam, UK), mouse monoclonal anti-MMP-9 (1:200; Santa Cruz Biotechnology, Santa Cruz, CA, USA), and anti-β-actin antibodies (1:5000; Merck, Darmstadt, Germany). Immunoreactive bands were identified as already reported [42].

### 4.5. Gene Expression Analysis

Total RNA was extracted using Total RNA Purification Kit (NORGEN Biotek) as previously described [47,48] and reported in Supplementary Materials. Quantitative real-time PCR (qPCR) was carried out using primer sequences for *IRF1*, *NFKb*, *TGRF2*, *CAAT*, *DOK2*, *RAS-GAP*, and *ACTB* genes. Primer sequences are listed in Supplementary Materials Table S1, as well as methods for RT-qPCR analysis. Relative gene expression was calculated by comparative Ct (ΔΔCt) method and converted to relative expression ratio (2−ΔΔCt) (Figure S1).

### 4.6. Statistics

Statistical analysis was performed using GraphPad Prism 6.0 software (GraphPad Software, San Diego, CA, USA). For immunophenotype data, results are expressed as median with interquartile range. Statistical differences were determined either using Mann–Whitney nonparametric *t*-tests between two groups with only one variable, or with Kruskal–Wallis non-parametric ANOVA with Dunn’s post-test for multiple variables. For Western blot and qRT-PCR analyses, individual values from independent densitometric measurements were summarized as means ± standard deviations (S.D.), and statistics were performed using one-way analysis of variance (ANOVA) followed by Tukey’s multiple comparison test. Values of *p* ≤ 0.05 were considered statistically significant.

## Supplementary Materials

The supporting information can be downloaded at: https://www.mdpi.com/article/10.3390/ijms232315165/s1. References [49,50,51,52,53] are cited in the supplementary materials.
