# Supplementary material for: CD200 as a Potential New Player in Inflammation during Rotator Cuff Tendon Injury/Repair: An In Vitro Model
Source: Int J Mol Sci. 2022 Dec 2;23(23):15165. doi: 10.3390/ijms232315165 (PMC9738060; doi:10.3390/ijms232315165)
Supplement: Supplementary file 1 [file ijms-23-15165-s001.zip › ijms-1985716-supplementary.pdf]

Supplementary Material

Total RNA from RCTCs treated and control cells after 24 h was extracted using the Total RNA Purification Kit (Cat. 17259, NORGEN Biotek), according to the manufacturer's instructions. Digestion of genomic DNA was carried out by DNaseI (Sigma) exposing the samples for 15 min at RT. Total RNA integrity was evaluated by 1% agarose gel electrophoresis with GelRed staining (Biotium). Then, total RNA was evaluated and quantified using a Thermo Scientific NanoDrop 2000c UV-Vis spectrophotometer at 260 nm and stored at −80°C until use. For the reverse transcription reaction (RT), 1 µg of total RNA from each sample was reverse transcribed into complementary DNA using Random Hexamer primers, dNTPs mix, and Tetro Reverse Transcriptase (Bioline) in a 20 µl reaction volume mixture, according to the manufacturer's instructions. cDNAs were diluted 3 times (60 µL final volume) and stored at -20° C until their use as template in RT-qPCR mixture. The RT-qPCR reaction consisted of 13 µL master mix containing SensiFAST TM SYBR Lo-ROX kit (Bioline, Tennessee, USA), primers, and 2 µL of the diluted cDNA in a total volume of 15 µL, according to manufacturer's instructions. The cycling program was two-step cycling protocol for 40 cycles (10 s at 95 °C for denaturation and 30 s at 60 °C for annealing/extension) with 7500 Fast Real-time PCR System (Life Technologies, NY, USA) followed by melt-profile analysis (7500 Software v2.3). For each qPCR analysis, each sample was performed in triplicate, and values were normalized to *ACTB* endogenous reference gene. Thus, quantitative PCR standard curves, amplifying different amounts of the same cDNA, were performed. The obtained cDNA was used as a template in the RT-qPCR mixture. In this experiment, 4 increasing concentrations of cDNA samples with a dilution factor of 1:4 have been prepared and analyzed with Quantitative real-time PCR (qPCR), using primer sequences listed in Supplementary Material Table S1. By plotting the log10 of the starting amount of cDNA (axis X) vs. the obtained Ct value (axis Y) of each sample, the efficiency of the primer couple has been demonstrated (see supplementary material Fig.S1). Efficient primers should give a proportional curve. As result, the slope of the regression curve for all primers was between -3,9 and -2.9 and the correlation coefficients were higher than 0.98 for most of them. Afterward, the efficiency of the primers ($\mathscr{E}$) was calculated following the formula reported below. The results have shown that all couples of primers were efficient (efficiency higher than 80%) and ready to be used for gene expression analysis.

$$\mathscr{E} = 100*(10^{-\frac{1}{Slop}} - 1)$$

The specificity of the qPCR product was verified using the melting curve analysis program.

Since a critical step required for data normalization in RT-qPCR analysis is the choice of reference genes (RG), the expression stability of three commonly used RGs (*GAPDH*, *ACTB*, and *CYPA)* was estimated using the BestKeeper software [1]. According to this software, *ACTB* was the most reliable RG in our experimental conditions, showing the lowest coefficient of variation and standard deviation (SD) values in all stimulated and control samples (data not shown). This result entirely agrees with a recently published paper, in which *ACTB* was the best suitable RG, among five commonly used RGs, in human tenocytes under either inflammatory or pro-fibrotic/healing stimulation [2].

1. Pfaffl MW. A new mathematical model for relative quantification in real-time RT-PCR. Nucleic Acids Res. 2001 May 1;29(9):e45. doi: 10.1093/nar/29.9.e45.
2. Ragni E, Perucca Orfei C, Bowles AC, de Girolamo L, Correa D. Reliable Reference Genes for Gene Expression Assessment in Tendon-Derived Cells under Inflammatory and Pro-Fibrotic/Healing Stimuli. Cells. 2019 Oct 1;8(10):1188. doi: 10.3390/cells8101188.

Gene	Primer sequence (5'–3')	Frag ment size (bp)	T melting (°C)	References
GAPDH	Forward: CCACTCCTCCACCTTTGACG<br>Reverse: CATGAGGTCCACCACCCTGT	115	64	(Klatte-Schulz et al. 2012)
CYPA	Forward: CTCGAATAAGTTTGACTTGTGTTT<br>Reverse: CTAGGCATGGGAGGGAACA	165	56-58	(Hellebrekers et al. 2006)
ACTB	Forward: CACCATTGGCAATGAGCGGTTC<br>Reverse: AGGTCTTTGCGGATGTCCACGT	135	62-64	Primer-Blast
IRF I	Forward: CAAATCCCGGGGCTCATCTGG<br>Reverse: CTGGCTCCTTTTCCCCTGCTTTG	160	63-64	(Cohen et al. 2014)
NF-kB	Forward: GCAGCACTACTTCTTGACCACC<br>Reverse: TCTGCTCCTGAGCATTGACGTC	130	61-62	ORIGENE
TGFBR2	Forward: GACAACGTCAGGTTCTGGCTCA<br>Reverse: CCGCCACTTTCCTCTCCAAACT	124	68	ORIGENE
CAAT/ EBPbeta	Forward: CACAGCGACGACTGCAAGATCC<br>Reverse: CTTGAACAAGTTCCGCAGGGTG	188	61-62	Primer-Blast
DOK2	Forward: GTACAGCAGCGCAGTCACAG<br>Reverse: AGCCCGGAGGGTATAGGAC	109	59-61	Primer-Blast
RAS-GAP	Forward: GGGACATCCAATAAACGCCTTCG<br>Reverse: TTTGCTACTTGGACACTATTCAGG	131	58-62	Primer-Blast

Table S1: Primer sequences and corresponding annealing temperatures for q-PCR analysis

Klatte-Schulz F, Pauly S, Scheibel M, Greiner S, Gerhardt C, Schmidmaier G, Wildemann B. Influence of age on the cell biological characteristics and the stimulation potential of male human tenocyte-like cells. Eur Cell Mater. 2012 Jul 12;24:74-89. doi: 10.22203/ecm.v024a06

Hellebrekers DM, Castermans K, Viré E, Dings RP, Hoebers NT, Mayo KH, Oude Egbrink MG, Molema G, Fuks F, van Engeland M, Griffioen AW. Epigenetic regulation of tumor endothelial cell anergy: silencing of intercellular adhesion molecule-1 by histone modifications.Cancer Res. 2006 Nov 15;66(22):10770-7. doi: 10.1158/0008-5472.CAN-06-1609.

Cohen S, Mosig R, Moshier E, Pereira E, Rahaman J, Prasad-Hayes M, Halpert R, Billaud JN, Dottino P, Martignetti JA. Interferon regulatory factor 1 is an independent predictor of platinum resistance and survival in high-grade serous ovarian carcinoma. Gynecol Oncol. 2014 Sep;134(3):591-8. doi: 10.1016/j.ygyno.2014.06.025.

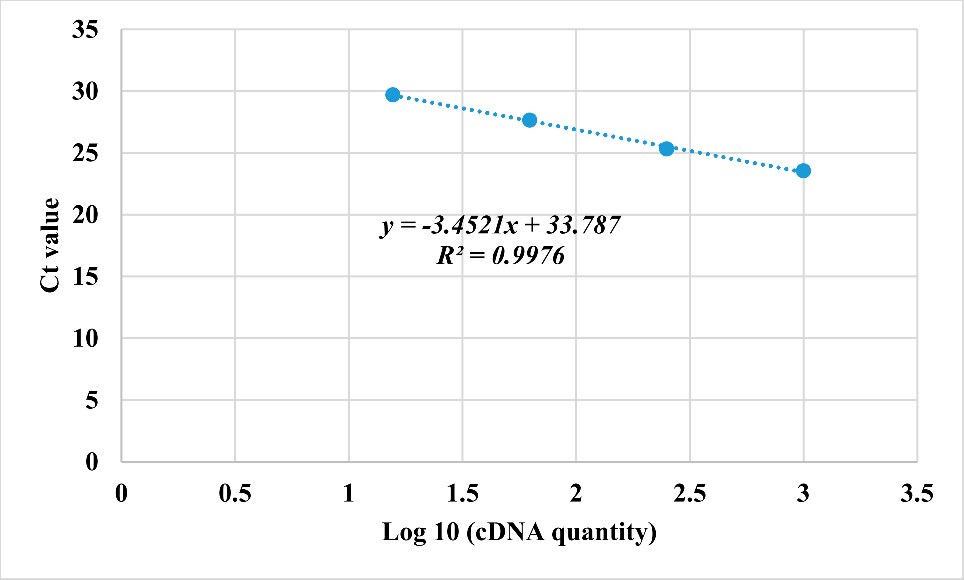

Figure S1: Example for the validation regression curve obtained for the *ACTB* gene.
